# Critical review of cancer mortality using hospital records and potential years of life lost

**DOI:** 10.1590/S1679-45082018AO4018

**Published:** 2018-04-06

**Authors:** Carolina Panis, Aedra Carla Bufalo Kawasaki, Claudicéia Risso Pascotto, Eglea Yamamoto Della Justina, Geraldo Emílio Vicentini, Léia Carolina Lucio, Rosebel Trindade Cunha Prates

**Affiliations:** 1 Group of Advanced Studies in Health Applied Sciences, Universidade Estadual do Oeste do Paraná, Francisco Beltrão, PR, Brazil

**Keywords:** Neoplasms/mortality, Hospital records, Potential years of life lost, Brazil, Neoplasias/mortalidade, Registros hospitalares, Anos potenciais de vida perdidos, Brasil

## Abstract

**Objective:**

To determine and discuss cancer mortality rates in southern Brazil between 1988 and 2012.

**Methods:**

This was a critical review of literature based on analysis of data concerning incidence and mortality of prostate cancer, breast cancer, bronchial and lung cancer, and uterine and ovarian cancer. Data were collected from the online database of the Brazil *Instituto Nacional de Câncer José Alencar Gomes da Silva*.

**Results:**

The southern Brazil is the leading region of cancer incidence and mortality. Data on the cancer profile of this population are scarce especially in the States of Santa Catarina and Paraná. We observed inconsistency between data from hospital registers and death recorded.

**Conclusion:**

Both cancer incidence and the mortality are high in Brazil. In addition, Brazil has great numbers of registers and deaths for cancer compared to worldwide rates. Regional risk factors might explain the high cancer rates.

## RESUMO

**Objetivo:**

Investigar e discutir os indicadores de mortalidade por câncer na Região Sul do Brasil entre 1988 e 2012.

**Métodos:**

Revisão crítica da literatura baseada na análise de dados referentes às estimativas de incidência e mortalidade dos cânceres de próstata, mama feminina, brônquios e pulmões, colo de útero e ovário, realizada por meio de consulta na base de dados online do Instituto Nacional de Câncer José Alencar Gomes da Silva.

**Resultados:**

A Região Sul lidera no país a incidência e a mortalidade das neoplasias estudadas. Há escassez de dados sobre o perfil do câncer nesta população, especialmente nos Estados de Santa Catarina e Paraná. Notou-se, ainda, incoerência entre os dados de registros hospitalares e registros de óbito no período estudado.

**Conclusão:**

Tanto a incidência quanto a mortalidade decorrentes dos cânceres estudados ainda são muito elevadas no Brasil, com significante número de registros da doença e de óbitos, quando comparado às taxas mundiais. Fatores de risco regionais podem explicar as elevadas taxas.

## INTRODUCTION

Cancer is one of the leading causes of death worldwide.^(^ ^1^ ^)^ From 2014 to 2015 Brazil had estimated more than 500,000 new cases of cancer,^(^ ^2^ ^)^ which placed the country among those with the highest cancer incidence in current days.^(^ ^3^ ^)^

Although the growing efforts for early screening and diagnosis, the associated risk factors for development of this disease are strongly present in Brazilian population, particularly smoking, occidental diet, obesity and sedentarism.^(^ ^4^ ^)^

**Figure 1:**
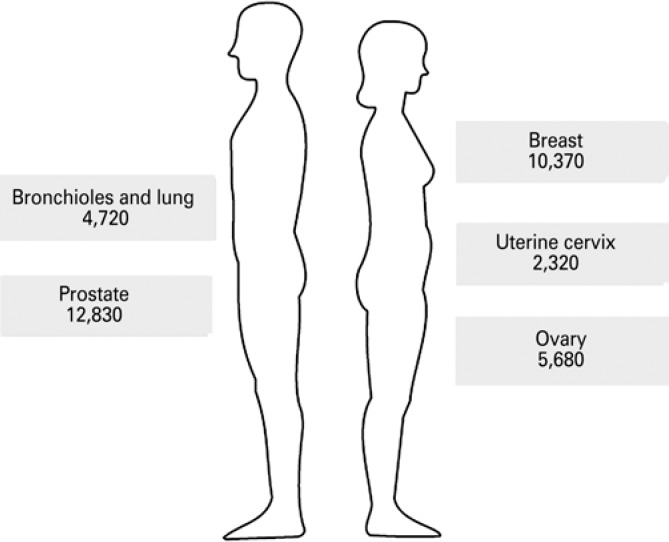
Estimated number of cancer cases selected by study in population of south Brazil in 2014. Data extracted from database of *Instituto Nacional de Câncer José Alencar Gomes da Silva*.^(^ ^2^ ^)^ Data collected for lung cancer, cancer of the trachea, and bronchial cancer, estimated by sum of new cases among men and women

A high cancer incidence in seen in most-populous and industrialized regions of the country; the south region. This region concentrates the highest incidence of neoplasias, according to figure 1, and this fact might be associated with southern population longer life expectancy and healthy habits.^(^ ^5^ ^)^ This high number of cancer cases among southern population still a reflex of great number of diagnoses and, consequently, increase records in official databases.^(^ ^6^ ^)^

Although the high cancer incidence and mortality rates in south Brazil, few studies have discussed possible associated factors with this disease. Some neoplasias such as ovarian and breast cancer have a strong genetic predisposition among population in this region prevalent.^(^ ^7^ ^)^ In addition, southern population high fat diet and consumption of smoked products favor the development of obesity, one of the main factors that lead to development of cancer in the modern society.^(^ ^8^ ^)^ Such factors contribute significantly to development of clinicopathological cancers with worse prognosis that may impact cancer morbidity and mortality.

## OBJECTIVE

To discuss the cancer issue in southern Brazil within the last 20 years based on information from the database of *Instituto Nacional de Câncer José Alencar Gomes da Silva* particularly issues related with notification of this disease and mortality related to prostate cancer, breast cancer, bronchial and lung cancer, and uterine and ovarian cancer.

## METHODS

This is a critical review on estimation analysis of incidence and indicators of mortality of prostate cancers, breast cancer, bronchial and lung cancer, uterine and ovarian cancer based on consultations of online database of National Cancer Institute José Alencar Gomes da Silva (INCA – *Instituto Nacional de Câncer*). Number of hospital records and deaths for each topography and by States of Southern Region were obtained from online data of hospital-based cancer registry of INCA (1998-2012), and from INCA's mortality atlas (2001-2012). Such registers were selected to compose this study because they included information that enabled to evaluate quality of service provide the hospital network in Brazil.

In addition, we evaluated data related with crude and adjusted rates and number of years of potential life lost for cancer from INCA's mortality atlas using based on total of Brazil population available in census from 2010 (https://mortalidade.inca.gov.br/MortalidadeWeb/pages/Modelo01/consultar.xhtml;jsessionid=CA2C390AB43798B3880F4078A4345436). Adjusted mortality rate by age evaluates the number of deaths in each age range regarding total of deaths in south population and this rate was directly standardized by primary source consulted in order to reduce bias that age factor can add to studies on cancer. Proportional mortality data were used to illustrate amount of deaths in population affected by cancer in studied period and number of years of potential life lost as indicator of total sum of years lost for each death because of cancer. Such associated indicators enabled to measure impact of each cancer in the studied population within each year.

All database mentioned were searched within the INCA website.^(^ ^2^ ^)^ The values presented were obtained from those calculated by databases, using as reference the estimated southern Brazilian population for each year. Coefficients were also adjusted by age by database searched. To discuss results, we used published studies in PubMed and Scientific Electronic Library Online (SciELO).

## RESULTS

Results obtained highlighted Southern Region as national leading region of cancer incidence and mortality from 1988 to 2012. This comparative analysis of number of hospital-register cancer between 2001 and 2012 for prostate cancer, breast cancer, bronchial and lung cancer, uterine and ovarian cancer showed expression values for Southern Region with special emphasizes to Rio Grande do Sul State, followed by Paraná and Santa Catarina (Table 1).

**Table 1:** Hospital-registers and deaths for cancer according to primary site incidence and categorization by States

			2001	2002	2003	2004	2005	2006	2007	2008	2009	2010	2011	2012
*Prostate*	*PR*	Registers	67	106	222	358	915	1,045	1,258	1,476	1,646	1,893	1,745	1,180
		Deaths	595	588	650	687	691	737	759	726	799	834	905	892
	*SC*	Registers	182	212	248	223	378	589	731	913	1,020	1,120	1,254	650
		Deaths	279	278	267	337	327	338	344	347	373	380	389	392
	*RS*	Registers	134	608	558	969	1,361	1,713	1,986	1,860	1,871	2,005	2,109	1,192
		Deaths	792	760	791	825	881	932	949	974	972	1,090	1,032	1,007
*Breast*	*PR*	Registers	98	198	335	481	1,173	1,224	1,731	1,912	2,665	2,656	2,141	1,814
		Deaths	538	584	541	569	619	630	642	684	722	729	803	821
	*SC*	Registers	465	445	465	400	648	973	1,231	1,681	1,823	1,733	1,768	964
		Deaths	264	293	296	283	324	320	344	346	400	435	470	491
	*RS*	Registers	313	1,617	1,499	1,828	2,334	2,687	3,251	2,641	2,780	2,573	2,893	1,507
		Deaths	872	893	952	980	945	1,022	1,029	1,020	1,045	1,136	1,147	1,104
*Bronchioles and lung*	*PR*	Registers	22	57	133	168	394	401	522	661	683	826	621	568
		Deaths	1,070	1,097	1,133	1,198	1,204	1,266	1,353	1,412	1,439	1,513	1,533	1,530
	*SC*	Registers	201	196	215	196	293	414	519	578	657	733	623	366
		Deaths	709	681	737	878	818	824	901	907	1,020	1,101	1,124	1,113
	*RS*	Registers	166	623	708	799	1,028	1,237	1,334	1,162	1,293	1,189	1,270	647
		Deaths	2,187	2,242	2,389	2,478	2,616	2,623	2,782	2,848	2,843	2,964	2,986	3,090
*Uterus*	*PR*	Registers	57	103	304	455	1,346	1,387	1,281	1,499	1,561	1,738	1,536	1,210
		Deaths	282	297	280	276	275	268	236	289	266	282	293	268
	*SC*	Registers	257	245	254	246	326	405	468	566	617	634	700	247
		Deaths	126	110	114	113	131	108	146	130	142	129	161	175
	*RS*	Registers	107	635	623	665	826	931	918	576	590	525	757	313
		Deaths	366	302	336	335	327	319	285	303	295	267	267	305
*Ovary*	*PR*	Registers	4	13	38	69	120	134	133	185	241	215	189	156
		Deaths	124	151	132	141	144	174	170	173	191	202	191	210
	*SC*	Registers	55	47	62	49	66	89	118	149	167	150	135	69
		Deaths	82	81	74	82	84	76	83	94	88	108	105	115
	*RS*	Registers	22	88	84	126	140	169	236	236	211	191	197	127
		Deaths	179	229	207	249	228	229	221	257	257	283	259	261

Prostate and breast cancer had higher incidence than number of deaths in Paraná and Rio Grande do Sul States. The same result was seen in Santa Catarina after 2004. In Southern Region, we also observed increase in registers of prostate cancer diagnosis, although this type of cancer did not show increase in mortality (Table 1).

Regarding bronchial and lung cancer, we observed an increase of number of hospital register and deaths, particularly in the State of Rio Grande do Sul. This was the leading State in number of deaths because of gynecologic cancers. The same result was seen in this State to the five cancer types analyzed.

Still, south region had the highest mortality rates for cancer. Table 2 shows that standardized world mortality rate was achieved and overpassed within the age 40 to 49 years, excepted to prostate cancer. Mortality rate increased gradually from 60 years of age, and higher indexes occurred among individuals aged 70 to 79 years (196.37/100,000) reaching 538.54/100,000 of those older than 80 years.

**Table 2:** Mortality, crude, adjusted rates by cancer per age, world and Brazilian population from 2010 per 100,000 men and women, according to primary site of men and women incidence

			Age range							Total in the period	Crude rate per region	Word standardized rate	Brazilian standardized rate
			20-29	30-39	40-49	50-59	60-69	70-79	≥80				
Prostate													
	Men	Number of deaths	13	22	244	1,838	7,508	14,968	13,361	37,980			
		Specific rate	0.02	0.05	0.65	7.26	47.79	196.37	538.54				
											12.35	15.37	18.42
Breast													
	Women	Number of deaths	306	2,743	7,663	10,179	8,889	6,930	4,708	41,451			
		Specific rate	0.56	5.61	19.76	37.85	49.94	70.70	116				
											13.17	13.32	14.29
Bronchioles and lung													
	Men	Number of deaths	175	674	4,384	14,205	24,131	20,141	6,991	70,812			
		Specific rate	0.32	1.43	11.76	56.13	153.61	264.24	281.79				
											23.02	28.20	29.46
	Women	Number of deaths	112	533	2,570	6,099	8,747	7,821	3,863	29,797			
		Specific rate	0.21	1.09	6.63	22.68	49.14	79.8	95.18				
											9.47	9.80	10.3
Uterus													
	Women	Number of deaths	499	2,304	3,948	3,906	3,078	1,945	906	16,600			
		Specific rate	0.92	4.71	10.18	14.52	17.29	19.84	22.32				
											5.27	5.27	5.66
Ovary													
	Women	Number of deaths	222	464	1,260	2,125	2,558	2,031	1,056	9,816			
		Specific rate	0.41	0.95	3.25	7.90	14.37	20.72	26.02				
											3.12	3.20	3.38

For breast cancer, 91.3% of cases of mortality occurred after 50 years, achieving rate of 116 cases/100,000 in age range ≥80 years. In relation to bronchial and lung cancer, highest rates of mortality were observed after 60 years, reaching 264.24/100,00 in men aged 70 to 79 years and 281.79/100,000 in men aged over 80 years.

Still this rate was lower among women, and relationship between sexes was 3.1 men for each women.

To each uterine cancer, we observed higher number of deaths among those aged 40 to 49 years. From this age range, this rate (10.18/100,000) was double of standardized Brazilian rate (5.66/100,000) and world rate (5.27/100,000), reaching 22.32/100,000 in age of 80 years. Similar results can be seen in death rates due to ovarian cancer in which age range from 60 to 69 years had 26.06% of deaths (14.37/100,000). From the 40 years, death rate increased significantly compared with age 30 to 39 years (0.95/100,000).

**Figure 2:**
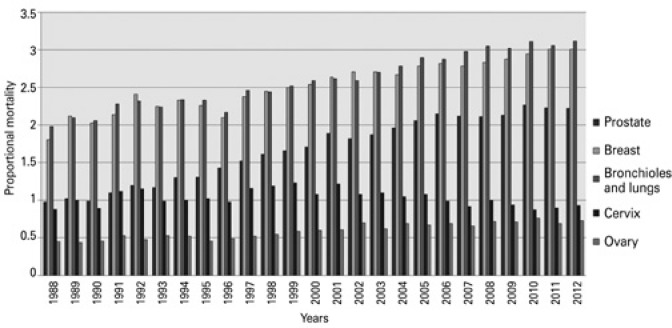
Non-adjusted proportional mortality per cancer

Source: Instituto Nacional de Câncer José de Alencar Gomes da Silva (INCA). Estimativa 2014 – Incidência de Câncer no Brasil. Rev Bras Cancerol. 2014;60(1):63-4.^(^^2^^)^

Non-adjusted proportional death rate (Figure 2) enabled to observe and analyze deaths of main cancers types (lung, breast, uterine, ovarian, and prostate) that occurred in south Brazil between 1988 and 2012. The association of data from Southern Region with hospital records showed that in 2003 a estimation of 4,980 prostate cancer cases occurred per 100,000 inhabitants in southern Brazil, however, the number found was 1,028. Although hospital register did not correspondent to incidence, the value obtained in hospital-based cancer register was lower than estimated. In 2012, however, the estimation by INCA was 9,490 new cases per each 100,000 inhabitants and, again, hospital registers showed a number about three times lower than expected, *i.e* 3,022 registers.

We observed a gradual increase of proportional mortality per breast cancer between 1988 to 2012. In case of bronchial and lung cancer death, there was an increase, with raising in rate from 1.98/100,000 in 1998 to 3.12/100,000 in 2012. Non-adjusted proportional mortality for uterine cancer had a decrease from 1.22% in 2001 to 0.93% in 2012. The non-adjusted proportional mortality rate for ovarian cancer showed an increase since 1996 compared with cancer death rate in Brazil.

**Figure 3:**
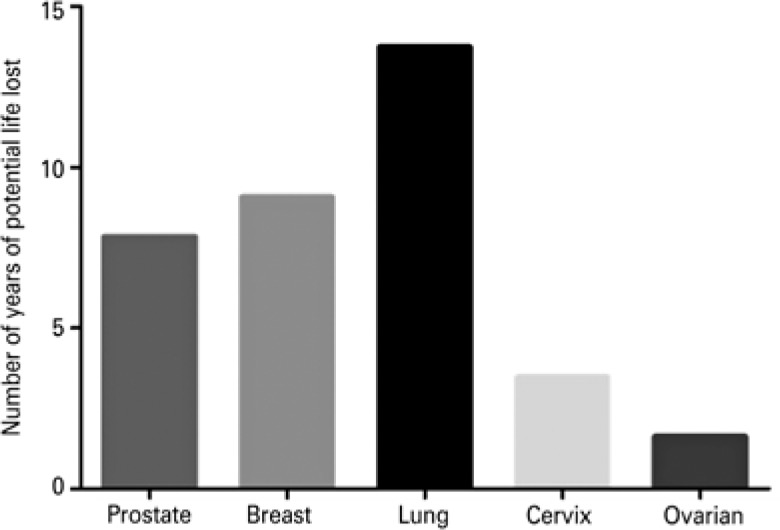
Mean potential years of life lost for cancer Estimation for breast cancer, uterine and ovarian cancer that was calculated for each 1,000 women and, for lung and prostate cancer, for each 1,000 inhabitants of south region between 1998 and 2012 from premise that high limit would be 80 years. Data calculated based on Brazilian population of 2010.

Source: Instituto Nacional de Câncer José de Alencar Gomes da Silva (INCA). Estimativa 2014 – Incidência de Câncer no Brasil. Rev Bras Cancerol. 2014;60(1):63-4.^(^^2^^)^

Figure 3 shows mean number of years of potential life lost because for cancer from 1998 to 2012 per each 1,000 inhabitants in Southern Region population aged no older than 80 years old.

Mean number of years of life lost by men and women diagnosed with bronchial and lung cancer appeared in all age ranges, and it was higher between 60 and 69 years, achieving 13.74 of potential years of life lost. In general, based on potential years of life lost indexes and potential years of life lost rate in relation to studied cancers, the prostate cancer revealed rate of 6.69, and for breast 9.08, bronchial and lung 13.74, uterine 3.49 and for ovarian cancer 1.9. Age range regarding number of deaths for prostate, bronchial and lung cancers was higher among those aged 60 to 69 years. Number of deaths for breast, uterine and ovarian cancer was higher among those aged 50 to 59 years. Women diagnosed with breast cancer in the analyzed period lost, on average, 9 years of life.

## DISCUSSION

Data presented in this study enabled to affirm that south region concentrated the highest national incidence of hospital register for cancer with high mortality rate in the studied period. Among analyzed markers, hospital cancer registers used as basis for our study are important to measure relevance of this disease in public health because they represent assistances yearly for each type of cancer in one or more institutions and it can enable calculation of incidence of any cancer.^(^ ^9^ ^)^ Mortality rate by age that evaluate the number of deaths in each age range regarding total of deaths in a specific period and among population living in specific geographic area,^(^ ^10^ ^)^ was higher compared with world rates. For this reason, it is important to highlight that prostate cancer, breast cancer, bronchial cancer, and lung cancer have higher mortality rates in Brazil, and high rate can be observed in south of the country. Aspects related to weakness of Brazilian system in cancer prevention, it diagnosis in advanced phase cases, population's habits and precarious life conditions, difficult to access health system and get screening^(^ ^11^ ^)^ can affect the observed numbers.

Southern Brazilian population has a good socioeconomic development profile that positively reflect prevention and early treatment of diseases.^(^ ^12^ ^)^ This aspects help to understand low crude mortality rate observed in data collected. Age seems to be the main risk factor for cancer because of increase in life expectation followed by increase of chronic-degenerative diseases such as cancer.^(^ ^13^ ^)^

Southern Region is highlighted because it presents better life expectations than the rest of the country throughout the years, and this represents an important fact to explain increased incidence of cancers registered in the study. This fact explain, *e.g.*, the high incidence of diagnosed prostate cancer, once longevity constitutes an important risk factor for development of this cancer.^(^ ^14^ ^)^

Cancers that were most common among men, we observed high number of prostate cancer records within this population in the studied period, although the predominance of European descent individuals. Southern Region has predominance of Caucasian population and prostate cancer has a higher incidence among African-descent individuals. This suggests that other regional factors, besides age, may influence high prevalence of this neoplasia in studied population with active investigation of prostate cancer by screening exams.^(^ ^15^ ^)^

Despite high incidence and high mortality rates by prostate cancer are relatively low compared with other types of cancers,^(^ ^14^ ^)^ mainly because this entails a relatively aggressive cancer.^(^ ^16^ ^)^

In bronchial and lung cancer the high number of registers and deaths can be closed correlated with smoking, mainly because this region has the highest prevalence of smokers in the country.^(^ ^17^ ^,^ ^18^ ^)^ This increase of mortality rate by bronchial and lung cancer is because of the growing number of women who smoke, and because of higher risk of development of different cancers.^(^ ^19^ ^)^ Mortality profile observed in bronchial and lung cancer in older population can still be associated with smoking history,^(^ ^19^ ^)^ once the incubation period of this pathology can last for 30 years.^(^ ^20^ ^)^ The reduced tendency of death among younger men probably reflect national actions to reduce smoking rates in Brazil.

Regarding cancer among women, the prevalence of smoking in the region is still an important risk factor to increase incidence of gynecology cancers, such as uterine cancer.^(^ ^2^ ^)^ Reduction of non-adjusted mortality observed in this study between 1999 and 2001 could be for gradual broad of screening services as well as strengthening and qualification of basic care network within last 20 years.

Age of ovarian cancer diagnosis ranged from 42.7 to 48 years, whereas mean described for sporadic cases is closed to 61 years old. These findings suggest possible existence of family heritage about one in each 10 cases of women with ovarian cancer that present standard that gives susceptibility for early occurrence of hereditary breast and ovarian cancer syndrome.^(^ ^21^ ^)^

In this sense, we observed in Rio Grande do Sul a high prevalence of breast cancer among young women. In this situation, this neoplasia is presented as aggressive disease of difficult treatment,^(^ ^22^ ^)^ which explain the high mortality rate observed after 50 years. Indeed, we observed that around 6% of southern population of metropolitan region have hereditary predisposition for breast cancer that commonly occurs in individuals younger 50 years and who present quite aggressive characteristics with high mortality rate.^(^ ^23^ ^)^

In addition, a study done by Cadaval Gonçalves et al., revealed that, between 1980 and 2005, Rio Grande do Sul had highest concentration in the country of death rates for breast cancer.^(^ ^24^ ^)^ Therefore, the study suggested that morbidity and mortality of the disease is a true fact in this population and it has been associated with socioenvironmental facts such as low formal education level, chronic exposition to agriculture products and overweight.^(^ ^25^ ^–^ ^27^ ^)^ The unclear data observed between incidence registers and number of deaths for cancer included in our study pointed out the need to standardize data register about cancer in Brazil.

Other indicators analyzed regarding mortality, the number of productive years of life lost, is an indicator that reflect total sum of years of potential life lost.^(^ ^28^ ^)^ However, the index called years of productive life lost enabled to compare different causes of death in a specific population.^(^ ^29^ ^)^ Both indexes allowed to measure the amount of years of life lost by population because of cancer. In this study, we observed that high-incidence cancers were also those that potentially debilitate population, and they reduced individuals’ years of life up to 14 years, which is case of individuals affected by lung cancer. Of note is that use of tobacco products, a risk factor to all studied cancers, often start during individuals’ adolescence. For this reason, a residue of this tobacco use is expected in more advanced age ranges, especially among men.

Great number of years of life lost for ovarian cancer is because of this disease aggressiveness and also because it is discovered in more advanced phases, which results in extremely low survival rate. On the other hand, in uterine cancer, we observed low rate of years of potential life lost compared with prostate cancer, breast cancer, and bronchial and lung cancer. This finding emphasizes the importance of oncological care programs in Brazil to promote women health that massive investments occurred since 2005 to implement a national oncological care plan.

Prostate cancer had a reduced impact on years of potential life lost compared with cancer of high incidence in south region. The number of years of life lost by men can have an important relationship with extended survival of patients with prostate cancer that can achieve up to 5 years after diagnosis and treatment.^(^ ^30^ ^)^

Based on mean life expectancy of Brazilian population that in 2010 was considered around 75 years old, we can affirm that almost one fifth of total years of individual's life is lost when people had suffered neoplasias such as bronchial and lung cancer. There is also the socioeconomic impact because of productive years of life lost, especially in cases of uterine cancer, because this latter is a potentially avoidable cancer. Our data suggest that these significant losses of productive years in Southern Region is a negative socioeconomic impact to Brazil.

## CONCLUSION

Both incidence and mortality of cancer are still high in Brazil with significant number of registers and deaths compare with worldwide rates. There is no agreement between number of hospital-based cancer registers and number of deaths because of cancer considering that, in some years, the register is lower than number of deaths. In addition, we observed a great number of death because of uterine cancer in south Brazil. Few studies were carried out to collected data on cancer profile in south Brazilian population, especially in States of Santa Catarina and Paraná.
